# Clinical significance of miR-129-5p in patients with neonatal sepsis and its regulatory role in the lipopolysaccharide-induced inflammatory response

**DOI:** 10.17305/bjbms.2020.5184

**Published:** 2020-12-29

**Authors:** Meng Li, Xiaoyang Huang, Qingcui Zhuo, Jinghui Zhang, Xiuli Ju

**Affiliations:** Department of Pediatrics, Qilu Hospital of Shandong University, Jinan, Shandong, China

**Keywords:** Neonatal sepsis, miR-129-5p, diagnosis, inflammatory response

## Abstract

Neonatal sepsis (NS) occurs in neonates within 28 days, especially preterm infants. The dysregulation of miRNAs is widely detected in NS. The study investigated the expression changes and clinical significance of miR-129-5p in NS patients and further explored the regulatory role of miR-129-5p in the lipopolysaccharide (LPS)-induced inflammatory response in monocytes. A total of 75 neonates with NS and 84 neonates without NS were recruited. Quantitative real-time polymerase chain reaction was used for the measurement of miR-129-5p expression. The receiver operating characteristic curve was constructed for diagnostic value analysis. ELISA was used to detect the concentration of inflammatory cytokines. Monocytes were isolated from the blood of neonates to investigate the role of miR-129-5p in the LPS-induced inflammatory response *in vitro*. miR-129-5p was low expressed in the serum of NS cases compared with controls. Serum miR-129-5p had a diagnostic value for NS with a sensitivity of 82.7% and specificity of 79.8%. There was close association for serum miR-129-5p with tumor necrosis factor (TNF)-α (r = −0.652, *p* < 0.001) and interleukins (IL-8) (r = −0.700, *p* < 0.001) levels in NS patients. Overexpression of miR-129-5p reversed the increasing trend of TNF-α and IL-8 induced by LPS, whereas miR-129-5p downregulation aggravated the increase of TNF-α and IL-8 induced by LPS in monocytes. MiR-129-5p was downregulated in the serum of NS patients, and it might be a promising biomarker for disease diagnosis. Overexpression of miR-129-5p alleviated the inflammatory response of NS.

## INTRODUCTION

Sepsis is a clinical syndrome, which is characterized by systemic inflammation, coagulopathy, and acute organ dysfunction due to an infection [1]. Neonatal sepsis (NS) is usually caused by bacteria, occurring in neonates within 28 days old [2]. NS contributes significantly to morbidity and mortality, especially in preterm infants [3]. The pathogenesis of NS is related to excessive inflammatory responses resulting in single or multi-organ dysfunctions [4]. Although several biological markers have been identified, such as C-reactive protein (CRP), interleukins (ILs), and procalcitonin (PCT), current strategies for the diagnosis of NS remain unsatisfactory as a result of their poor specificity [5,6]. Therefore, improvement in the diagnosis of NS is still urgent, which will be beneficial for the appropriate interventions as early as possible to improve survival outcomes.

During the past decades, a series of differentially expressed microRNAs (miRNAs) have been detected in various human diseases, and their clinical role in disease diagnosis and prognosis attracts more and more attention [7,8]. MiRNAs are small non-protein-coding RNA molecules with a length of 18–25 nucleotides and have been suggested to be involved in the regulation of a series of basic cellular processes such as differentiation, proliferation, apoptosis, and autophagy [9,10]. It has been reported that miRNAs can regulate the inflammatory pathways related to various human diseases, including NS [11,12]. Dysregulation of miRNAs is common occurred in NS and plays great significance for disease diagnosis such as miR-181a, miR-150, and miR-146a [11,13,14]. The functional significance of miR-129-5p in immune response has been investigated widely in recent years [15,16]. Notably, Wang et al. reported that miR-129-5p was down-regulated in septic mice through microRNA microarray and quantitative real-time polymerase chain reaction (qRT-PCR) [17]. However, limited information concerning the clinical value and biological function of miR-129-5p in NS has been reported.

In the current study, we aimed to investigate the expression changes and clinical significance of miR-129-5p in NS patients. Additionally, we further explored the regulatory role of miR-129-5p in the lipopolysaccharide (LPS)-induced inflammatory response in monocytes.

## MATERIALS AND METHODS

### Study population and sample collection

In the current study, 75 cases with NS were recruited who were admitted to Qilu Hospital of Shandong University from June 2015 to March 2019. The NS patients were diagnosed according to the criteria established at the 2003 Kunming Neonatal Sepsis Definitions Conference [18]. Serum samples were collected from the patients at the time of initial laboratory evaluation. Additionally, another 84 neonates diagnosed with respiratory infection or pneumonia in the same hospital were recruited as control groups. Neonates in the control group had no any symptoms and signs of sepsis. The sample size estimation was based on preliminary data, and calculated using a two independent proportions power analysis with alpha = 0.05 and a power of 90% (beta=0.1). Moreover, results indicated that 68 individuals are needed for each group. Demographics and clinical characteristics of the study population were recorded in Table 1.

**TABLE 1:**
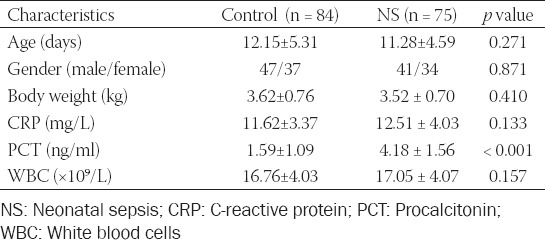
Clinical characteristics of the study population

### Cell culture and transfection

As the previous study described, the monocytes were isolated using density gradient centrifugation with FicollPaque (Amersham Pharmacia, Biotech AB) [19]. The specific cell markers CD14 and CD45 were used to evaluate the purity of the cells, and the purity of the cells was confirmed to be more than 95% by flow cytometry. The extracted monocytes were incubated in RPMI-1640 medium (Gibco; Thermo Fisher Scientific, Inc.) supplemented with 10% fetal bovine serum (Gibco; Thermo Fisher Scientific, Inc.) at 37°C. To mimic the inflammatory condition of NS *in vitro*, the monocytes were stimulated by 100 ng/ml LPS (Sigma-Aldrich; Merck KGaA) for 4 h.

Monocytes were plated into 48-well plates and lipofectamine 2000 (Thermo Fisher Scientific, Inc.) was used for the transfection according to the manufacturer’s protocols. miR-129-5p mimic (5’-CUUUUUGCGGUCUGGGCUUGC-3’), miR-129-5p inhibitor (5’-GCAAGCCCAGACCGCAAAAAG-3’), and the negative control (miR-NC, 5’-CAGUACUUUUGUGU AGUACAAA-3’) were purchased from Shanghai GenePharma Co., Ltd. (Shanghai, China).

### RNA extraction and quantitative real-time polymerase chain reaction (qRT-PCR)

According to the manufacture’s protocol, total RNAs were extracted using the TRIzol kit (Invitrogen, Carlsbad, CA, USA). Then, the miScript Reverse Transcription Kit (QIAGEN, Germany) was used for the reverse transcription according to the introductions. The qRT-PCR procedures were performed using the SYBR Green PCR Kit (Takara, Otsu, Shiga, Japan). The relative expression of miR-129-5p was calculated using the comparative delta CT (2^−DDCt^) method and normalized to U6. The primer sequences were as follows: miR-129-5p forward: 5’-ACACTCCTTTTTGCGTCTGGGCTTGC-3’ and reverse: 5’-TGGTGTCGTGGAGTCG-3’; U6 forward: 5’-TTACATTGCTATCCACAGAACGG-3’ and reverse 5’- CTATGCTGCTGCTTTTTGCTC-3’.

### Measurement of cytokines

ELISA was used to detect the concentration of the inflammatory cytokines tumor necrosis factor (TNF)-α and IL-8 according to the manufacturer’s protocol.

### Ethical statement

This study design was approved by the Ethics Committee of Qilu Hospital of Shandong University, and written informed content was collected from the guardian of each participant.

### Statistical analysis

SPSS version 18.0 software (SPSS Inc., Chicago, IL) and GraphPad Prism 5.0 software (GraphPad Software, Inc., USA) were used for data analysis. The data were presented as the mean ± standard deviation. Differences between groups were compared using Student’s t-test for continuous variables, the χ^2^ test for categorical variables. The difference between multiple groups was compared using one-way analysis. Pearson’s analysis was used for correlation analysis. Receiver operating characteristic (ROC) curve was constructed to evaluate the specificity and sensitivity of miR-129-5p for the diagnosis of NS. *p* < 0.05 was considered to be statistically significant.

## RESULTS

### Demographics of the study population

In the current study, a total of 75 cases with NS and 84 controls were included. It was found that there is no significant difference in age, gender, body weight, CRP, and WBC between NS and the control group (*p* > 0.05, Table 1). However, NS cases had a significantly high level of PCT compared with the control group (*p* < 0.001).

### Serum level of miR-129-5p in NS patients

**FIGURE 1:**
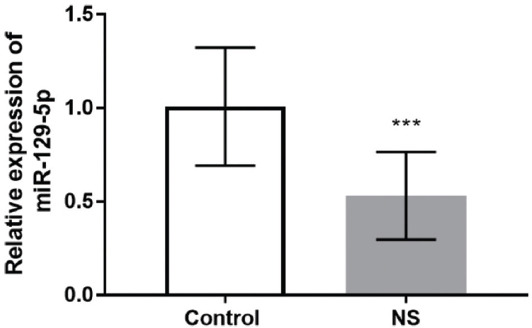
MiR-129-5p was low expressed in the serum of neonatal sepsis compared with the control group. *** p < 0.001.

QRT-PCR was performed to detect the level of miR-129-5p in the serum of NS. As shown in Figure 1, miR-129-5p was low expressed in the serum of NS compared with the control group (*p* < 0.001). The results indicated that miR-129-5p might be involved in the development of NS.

### Diagnostic value of miR-129-5p for NS

**FIGURE 2:**
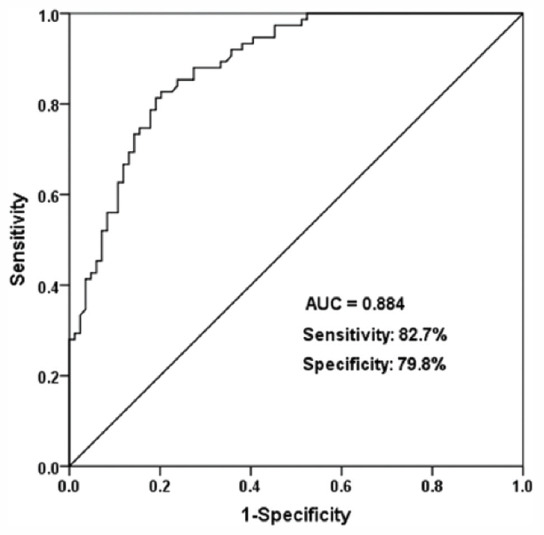
A receiver operating characteristic curve was established to evaluate the diagnostic value of miR-129-5p for neonatal sepsis. The AUC was 0.884, with the sensitivity of 82.7% and specificity of 79.8% at the cutoff value of 0.734.

According to the serum levels of miR-129-5p in NS patients and controls, a ROC curve was established to evaluate the diagnostic value of miR-129-5p for NS. As shown in Figure 2, the AUC was 0.884, with the sensitivity of 82.7% and specificity of 79.8% at the cutoff value of 0.734. The results suggested the diagnostic value of miR-129-5p for NS.

### Association of serum miR-129-5p with TNF-α and IL-8 levels in NS patients

**FIGURE 3:**
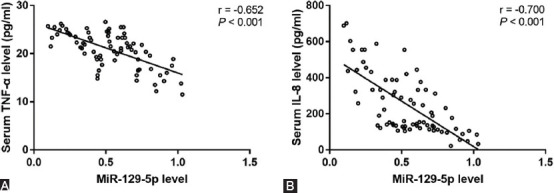
Correlation of miR-129-5p with IL-8 and tumor necrosis factor (TNF-α) levels in neonatal sepsis (NS) patients. There was close association for serum miR-129-5p with TNF-α (r = −0.652, p < 0.001) and interleukins-8 (r = −0.700, p < 0.001) levels in NS patients.

Considering the exaggerated inflammatory response in NS patients, we further explored the correlation of miR-129-5p with IL-8 and TNF-α levels in NS patients. As shown in Figure 3, there was close association for serum miR-129-5p with TNF-α (r = −0.652, *p* < 0.001) and IL-8 (r = −0.700, *p* < 0.001) levels in NS patients. These results demonstrated the potential role of miR-129-5p in inflammatory responses for NS.

### Overexpression of miR-129-5p alleviates pro-inflammatory responses in monocytes

**FIGURE 4:**
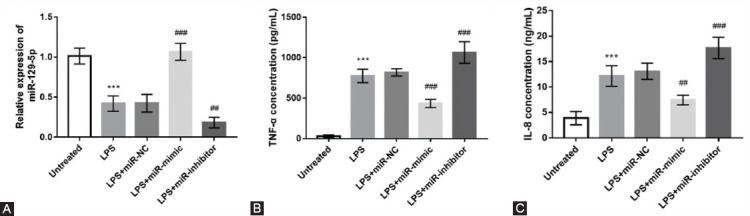
Overexpression of miR-129-5p alleviates pro-inflammatory responses in monocytes. (A) miR-129-5p mimic transfection significantly increased the level of miR-129-5p compared with the lipopolysaccharide (LPS) treated cells, reversely, miR-129-5p inhibitor transfection significantly aggravated the decrease of miR-129-5p. (B, C) Overexpression of miR-129-5p reversed the increasing trend of tumor necrosis factor (TNF-α) and interleukins (IL-8) induced by LPS, whereas miR-129-5p downregulation aggravated the increase of TNF-α and IL-8 induced by LPS. *** p < 0.001, compared with untreated group; ^#^p < 0.05, ^##^p < 0.01, ^###^p < 0.001.

Considering the close association of serum miR-129-5p with TNF-α and IL-8 levels in NS patients, we further explored the regulation of miR-129-5p on inflammatory cytokines in monocytes. As shown in Figure 4A, after LPS treatment, miR-129-5p level was decreased significantly in monocytes, which was consistent with the results observed in the serum. In addition, the concentration of TNF-α and IL-8 was also increased significantly by LPS treatment (Figure 4B and C).

Then, cell transfection was performed to regulate the expression of miR-129-5p in monocytes. It was observed that miR-129-5p mimic transfection significantly increased the level of miR-129-5p compared with the LPS treated cells, reversely, miR-129-5p inhibitor transfection significantly aggravated the decrease of miR-129-5p (Figure 4A). In addition, we also found that overexpression of miR-129-5p reversed the increasing trend of TNF-α and IL-8 induced by LPS, whereas miR-129-5p downregulation aggravated the increase of TNF-α and IL-8 induced by LPS (Figure 4B and C).

## DISCUSSION

NS leads to high mortality in neonates and may further bring long-term negative consequences. Accurate diagnosis of NS is important for timely disease treatment. NS is generally diagnosed according to the combination of clinical presentation and laboratory diagnosis, including cultures and infection markers, such as CRP, PCT, WBC, and so on [20,21]. However, the diagnosis of NS can be delayed because of the nonspecific clinical symptoms and infection markers. Therefore, the exploration of new biomarkers is urgent for the early diagnosis of NS, which could improve the clinical prognosis.

In recent years, numerous studies focus on the diagnostic or prognostic values of miRNAs in various human diseases, including NS. For example, aberrant miR-181a was supported to be a non-invasive diagnostic biomarker for NS by Liu et al., and overexpression of miR-181a alleviated the inflammatory response [11]. Another study reported the dysregulation of miR-15a/16 in NS patients, and its diagnostic value for NS was detected by the ROC analysis [22]. Pneumonia/respiratory tract infection in neonates increases the risk of NS, and identifying NS cases from cases with pneumonia/respiratory tract infection is of great significance for the efficient treatment of disease and improvement of clinical outcome [23]. Therefore, in the present study, neonates who suffered from respiratory infection or pneumonia were recruited as the control group, because blood samples were difficult to obtain from healthy individuals for ethical considerations. Nevertheless, neonates with infections, but not sepsis, already underwent blood collection and examination, and their blood samples were available with the approval from the families. Additionally, infections in neonates with pneumonia/respiratory tract infection contribute to the occurrence of NS [24]. Therefore, it is necessary to explore early diagnostic biomarkers to screen the NS cases from the infection cohort, which is significant for the early diagnosis and prevention of NS. The qRT-PCR results indicated that miR-129-5p was down-regulated in NS patients compared with the control group. Consistently, miR-129-5p has also been reported to be at low expression in septic mice through microRNA microarray and qRT-PCR [17], which supported our present results. Considering the dysregulation of miR-129-5p in NS patients, we further explored its diagnostic value for NS. The ROC analysis results indicated that serum miR-129-5p might be a promising biomarker for NS.

Sepsis in neonates is characterized by the persistence and prevalence of pro-inflammatory mediators. Elevated levels of pro-inflammatory cytokines such as TNF-α and IL-8 have been widely detected in both animal models and infants suffering from NS [5,24]. Seriously, the uncontrolled pro-inflammatory responses contribute to the high morbidity and mortality in NS. Thus, it is of great significance to examine the underlying mechanism of the exaggerated inflammatory response in NS patients. In the present study, according to the serum levels of miR-129-5p, IL-8, and TNF-α in NS patients, a close association was detected for serum miR-129-5p with TNF-α and IL-8. These results demonstrated the potential role of miR-129-5p in inflammatory responses for NS. Previously, the deficiency in miR-129-5p was demonstrated to exacerbate inflammation in several diseases. In a study about spinal cord injury (SCI), miR-129-5p was detected to be downregulated in spinal tissues of mice after SCI, and overexpression of miR-129-5p was suggested to alleviate SCI induced secondary injury through suppressing the inflammatory response [25]. In addition, miR-129-5p was reported to be involved in the regulation of lncRNA MALAT1 in the inflammatory response of microglia [26]. This evidence indicates the anti-inflammatory role of miR-129-5p in different human diseases. In the present study, downregulation of miR-129-5p was also identified in NS patients and was negatively associated with the elevation of pro-inflammatory cytokines.

As previous evidence reported, multiple cell types participate in the innate immune response, and monocytes and macrophages cells are considered to be the primary cells involved in neonatal immune response [27]. In the current study, we further investigated whether the deficiency in miR-129-5p is responsible for the increased inflammatory response in monocytes. First, the monocytes were stimulated by LPS to mimic the inflammatory of NS *in vivo*, it was found that miR-129-5p level was decreased significantly in monocytes after LPS treatment, which was consistent with the results observed in the serum of NS patients. In addition, through cell transfection, overexpression of miR-129-5p was proved to reverse the elevation of TNF-α and IL-8 induced by LPS. These results suggested the anti-inflammatory effects of miR-129-5p in NS patients. However, the animal studies are not included in the present study, which will be beneficial to verify the present results. Additionally, the underlying mechanism of the role of miR-129-5p in NS should be further investigated in future studies.

## CONCLUSION

In conclusion, the present results indicated the downregulation of miR-129-5p in the serum of NS, and the dysregulation of miR-129-5p might be a promising biomarker for disease diagnosis. Additionally, overexpression of miR-129-5p alleviated the inflammatory response of NS. These results provide a theoretical basis for the diagnosis and treatment of NS.
